# Research Hotspots, Trends, and Molecular Mechanisms of Diabetes and Sarcopenia: A Bibliometric and Bioinformatics Analysis

**DOI:** 10.2174/0118715303427280251205051848

**Published:** 2026-06-05

**Authors:** Xuwu Chen, Zijing Li, Tanyue Zhou, Wenjuan Wu, Long Chen, Xiangyang Chen, Maoyuan Wang

**Affiliations:** 1 Department of Rehabilitation Medicine, The First Affiliated Hospital of Gannan Medical University, Ganzhou City, Jiangxi Province, 341000, China;; 2 Rehabilitation College of Gannan Medical University, Ganzhou City, Jiangxi Province, 341000, China;; 3 School of Basic Medical Sciences, Gannan Medical University, Ganzhou City, Jiangxi Province, 341000, China;; 4 The First Clinical Medical College of Gannan Medical University, Ganzhou City, Jiangxi Province, 341000, China

**Keywords:** Diabetes, sarcopenia, bibliometric, bioinformatics, CiteSpace, molecular targets

## Abstract

**Introduction:**

The co-occurrence of diabetes mellitus (DM) and sarcopenia is increasingly recognized as a significant clinical challenge, yet the underlying molecular mechanisms and research landscape remain incompletely characterized.

**Methods:**

Relevant literature was retrieved from the Web of Science database, and bibliometric visualization was conducted using CiteSpace 6.2.R4 and VOSviewer 1.6.20. Genes associated with both diseases were obtained from the GeneCards and OMIM databases, and a protein–protein interaction network was constructed. Kyoto Encyclopedia of Genes and Genomes (KEGG) and Gene Ontology (GO) functional enrichment analyses were performed. Bibliometric findings were cross-validated with molecular mechanisms to identify convergent evidence.

**Results:**

The number of publications and citation frequency in this field showed a significant upward trend. China, Japan, and the United States contributed 58.6% of research papers, with Kyoto Prefectural University of Medicine, Seoul National University, and other institutions as major contributors. Fukui Michiaki was the most productive author, and Nutrients was the primary journal. Research themes evolved from early studies on molecular pathological mechanisms to investigations of clinical phenotypes and complications, and further to nutritional interventions, exercise therapy, and population management. Bibliometric analysis identified “insulin resistance” as the second most frequent keyword (212 occurrences), correlating with bioinformatics identification of INS as the primary hub gene among 97 shared targets. Five core genes (INS, ALB, AKT1, IL6, TNF) were significantly enriched in PI3K/MAPK and inflammatory pathways, supporting bibliometric emphasis on chronic inflammation.

**Discussion:**

Integration of bibliometric and bioinformatics results highlights insulin signaling, chronic inflammation, and nutritional status as common mechanisms linking DM and sarcopenia. These findings support the translational potential of lifestyle interventions (nutrition and resistance exercise) and insulin-sensitizing or anti-inflammatory therapies. Future research should focus on experimental validation and the development of combination strategies targeting the identified molecular network.

**Conclusion:**

This study constructed a comprehensive knowledge map for diabetes and sarcopenia research, systematically elucidating current research status, hot topics, and molecular mechanisms. The findings provide a scientific basis for developing joint prevention and treatment strategies and guiding future research directions.

## 1 INTRODUCTION

Diabetes mellitus (DM) is a group of metabolic diseases characterized by chronic hyperglycemia, primarily resulting from metabolic disorders caused by defective or impaired insulin secretion [1]. DM can be broadly categorized into type 1 diabetes mellitus (T1DM, autoimmune insulin deficiency), type 2 diabetes mellitus (T2DM, predominated by insulin resistance), and gestational diabetes mellitus [2]. Sarcopenia is a progressive skeletal muscle disease marked by the triad of reduced muscle mass, diminished muscle strength, and impaired physical performance [3]. It is often accompanied by chronic inflammation, metabolic diseases (*e.g*., DM, cardiac and renal disorders), and malnutrition, all of which significantly increase the risk of falls and mortality [4, 5].

Both conditions are major global health challenges, particularly in aging populations where their prevalence continues to rise, imposing substantial medical and socioeconomic burdens [4-6]. Growing clinical and epidemiological evidence indicates that DM and sarcopenia are not independent conditions but exhibit significant comorbidity and complex pathophysiological interactions [5, 7-9]. Patients with T2DM have a markedly increased risk of sarcopenia, while the muscle mass loss and metabolic dysfunction associated with sarcopenia can exacerbate insulin resistance and impair glycemic control, creating a vicious cycle [10-12].

This close clinical association suggests shared molecular mechanisms between the two diseases. Existing studies propose that chronic inflammation (mediated by factors such as TNF-α and IL-6), impaired insulin signaling pathways (*e.g*., PI3K/AKT), and oxidative stress from mitochondrial dysfunction may be key links connecting DM-related muscle metabolic abnormalities with muscle loss in sarcopenia [8, 13, 14]. Concurrently, research in this field has been rapidly expanding, yet the distribution of studies, knowledge structure, and development trends remain unclear—information critical for understanding the field and guiding future research directions.

Bibliometrics, which applies statistical methods to quantitatively analyze scholarly publication data, has become widely accepted for evaluating academic output and identifying emerging research trends [15, 16]. When combined with bioinformatics approaches that elucidate molecular mechanisms, this integrative method can assess whether research hotspots align with underlying biological pathways. Such convergence validation is vital for identifying therapeutic targets and guiding research priorities. In this study, we employ an innovative approach that integrates bibliometric analysis of global research trends with bioinformatics identification of shared molecular mechanisms. By cross-validating research hotspots with molecular evidence, we aim to construct a comprehensive knowledge framework bridging scientific trends with biological mechanisms, ultimately informing evidence-based therapeutic strategies for DM–sarcopenia comorbidity.

## 2 MATERIALS AND METHODS

### 2.1 Bibliometric Analysis

#### 2.1.1 Data Source and Search Strategy

The data were retrieved from the Web of Science Core Collection (WOSCC), widely regarded as the leading database for bibliometric studies due to its comprehensive coverage and rich citation information, which enables precise identification of research trends and emerging focus areas within specialized fields [17]. The detailed workflow is illustrated in Fig. (**1**). The search strategy employed was: TS = (sarcopenia) AND TS = (“diabetes mellitus” OR “diabetes”), with a time span from 2015 to 2024.

#### 2.1.2 Inclusion and Exclusion Criteria

Inclusion criteria: (1) papers included in WOSCC from 2015 to 2024; (2) research papers related to the topic of DM and sarcopenia.

Exclusion criteria: (1) papers not related to the topic of DM and sarcopenia; (2) non-academic articles, such as conference papers, news reports, book chapters, *etc*.; (3) duplicated publications; (4) articles not in English.

#### 2.1.3 Data Processing and Analysis

Two researchers independently evaluated the retrieved literature using predetermined inclusion and exclusion criteria. Initial screening involved reviewing article titles and abstracts for direct relevance to the study topic, with clearly ineligible materials excluded. Discrepancies were resolved through consensus discussions, and a third researcher adjudicated unresolved cases to maintain objectivity. Ultimately, 1,064 documents were extracted from WOSCC citation-referenced text files, providing comprehensive data on authors, keywords, journals, countries, institutions, and references.

Bibliometric analysis and visualization were conducted using CiteSpace 6.2.R4 [18] and VOSviewer 1.6.20 [19] to interpret topic trends, while Excel was used for descriptive analysis of publication parameters, including countries, years, and journals. CiteSpace 6.2.R4 parameters were configured as follows: time slicing (1 year per slice), years per slice (1), term sources (title, abstract, author keywords, and keywords plus), and node types (reference, author, keyword, institution, country). VOSviewer 1.6.20 parameters included: minimum number of occurrences (5 for keywords, 5 for citations), resolution (1.0), and minimum cluster size (1).

Centrality metric explanation: Betweenness centrality (BC) quantifies a node's importance as a bridge within a network. A higher BC value indicates that the node exerts greater influence in connecting different research communities and facilitating knowledge transfer.

#### 2.1.4 Disease Target Gene Screening

Potential targets associated with sarcopenia and DM were obtained by integrating the GeneCards database [20] (https://www.genecards.org/) and the OMIM database [21] (https://omim.org/): “diabetes” and “sarcopenia” were used as keywords to search the GeneCards database, and retained targets with a relevance score ≥ 10 to ensure reliability. The OMIM database was simultaneously searched to supplement the associated targets. After merging the target sets from the two databases, duplicate entries were removed, and the species was restricted to human genes (*Homo sapiens*). The final disease target dataset was then used for subsequent analysis.

#### 2.1.5 Construction of PPI Networks and Functional Enrichment Analysis

Using the Venn diagram platform (https://www.bioinformatics.com.cn/), generate a Venn diagram to determine the intersection genes between the two disease target sets. Submit the intersection genes to the STRING database (https://cn.string-db.org/) (parameter settings: Homo sapiens species restriction, confidence score ≥ 0.4 (medium confidence), hide free nodes) to construct a protein-protein interaction (PPI) network. The resulting network data were imported into Cytoscape (version 3.8.0) for topological property analysis (degree centrality to screen core genes) and for visualization modification. Degree centrality calculation using the NetworkAnalyzer plugin, where degree represents the number of direct connections for each node. Network visualization employed a circular layout with node size proportional to degree values and edge thickness representing interaction confidence. Functional enrichment analysis was performed using the DAVID database (https://davidbioinformatics.nih.gov/) (Gene Ontology biological processes/molecular functions/cellular components, KEGG pathway analysis) to identify significantly enriched entries (Benjamini-Hochberg corrected *p*<0.05), with final results visualized using bubble charts/bar charts.

## 3 RESULTS

### 3.1 Annual Publication Trends in DM and Sarcopenia

Excel was used to construct the trend in annual publications on DM and sarcopenia. Fig. (**2**) shows that the number of publications gradually increased from approximately 30 in 2015 to a peak of 250 in 2023, indicating a sustained upward trend. The number of citations rapidly grew from an extremely low level in 2015 to approximately 10,000 in 2024, with particularly noticeable acceleration after 2020. The R^2^ value in the linear equation is 0.9401, indicating a strong linear correlation between citation frequency and time.

### 3.2 Distribution of Publications by Country and Institution

A total of 63 countries with 307 institutions have been studied regarding the link between DM and sarcopenia. One thousand sixty-four publications from different countries were analyzed using VOSviewer, with a minimum publication count of 5. The figure shows the network and density view of the connections between the countries (Figs. **3A** and **B**). Table **1** presents the top 5 countries by publication output: China, Japan, the United States, South Africa, and Italy. China leads with the highest number of publications (294 articles, 5,202 citations, total link strength of 63), followed by Japan (199 articles) and the United States (130 articles). Notably, the United States demonstrates the highest citation impact with 8,488 citations and the strongest collaborative network with a total link strength of 122. These three leading countries collectively account for 58.6% of total publications, demonstrating their dominant position and sustained influence in DM and sarcopenia research.

As shown in Table **2**, Kyoto Prefectural University of Medicine has the highest number of publications, followed by Seoul National University and Yonsei University with 26 and 25 publications, respectively, reflecting the leading positions of these institutions in the fields of DM and sarcopenia. The high centrality of Hospital Universitario de Getafe not only highlights its key position as a link in institutional collaboration (Table **3**), but also underscores its indispensable role in promoting and coordinating international cooperation.

### 3.3 Author Analysis

A total of 378 authors contributed to the publications, Table **4** lists the top 5 authors with the number of publications. Fukui Michiaki emerges as the most prolific author with 26 publications and extensive collaborative networks. In one of his notable publications, Fukui Michiaki demonstrates that DM accelerates sarcopenia development through multiple pathological mechanisms, including hyperglycemia, insulin resistance, inflammation, oxidative stress, increased muscle-inhibitory factors, and mitochondrial dysfunction [22]. This comprehensive mechanistic framework explains the significantly increased prevalence of sarcopenia observed in diabetic patients, highlighting the complex interplay between these two conditions.

### 3.4 Analysis of Journals

A total of 411 journals published articles on this topic. Table **5** lists the top five journals by number of publications, with nutrients leading with 40 papers, indicating its prominence in this research field. One article in nutrients explored the vicious cycle between T2DM and skeletal muscle atrophy: in T2DM patients, insulin resistance reduces muscle glucose uptake and accelerates muscle loss, while the resulting muscle mass reduction further impairs glucose metabolism, exacerbating hyperglycemia. Hyperglycemia, in turn, causes mitochondrial dysfunction, chronic inflammation, and oxidative stress, which further impair muscle function and promote muscle deterioration [23].

Other highly productive journals include BMC Geriatrics, Journal of Cachexia, Sarcopenia and Muscle, Journal of Clinical Medicine, and Journal of Diabetes Investigation, covering topics ranging from basic science to clinical applications. VOSviewer was used to identify journals with significant co-citation frequency in this field, with the minimum number of articles set to 5; the results are shown in Figs. (**4A** and **B**). Analysis of the cited journals, based on node size, network connections, and density, revealed that Nutrients, Journal of Cachexia, Sarcopenia and Muscle, Journal of Diabetes Investigation, and PLoS ONE are the most prominently co-cited journals, reflecting substantial research attention on DM and sarcopenia.

The journal dual-map overlay illustrates citation relationships across disciplines (Fig. **4C**). Nodes are connected by curved streamlines representing knowledge transfer through citations. The three thickest stripes highlight core citation pathways: the orange stripe shows that articles published in molecular/biological/immunology journals related to sarcopenia and DM are frequently cited by molecular/biological/genetics journals, while the two green stripes indicate that literature from medicine/medical/clinical journals is often cited by health/nursing/medicine and molecular/biology/genetics journals. Analyzing these journals and their citation patterns provides insight into authors’ preferred outlets and offers valuable references for future research.

### 3.5 Citation Analysis of Reference Literature

Analyzing cited references helps reveal the knowledge base of a research field, as heavily cited literature is generally influential and highly credible. Using CiteSpace to generate a citation-based network in the field of DM and sarcopenia (Table **6**), Cruz-Jentoft AJ's “Sarcopenia: Revised European Consensus on Definition and Diagnosis” [24] emerged as the most cited article. This work updates the 2010 consensus on sarcopenia, revising its definition and diagnostic criteria based on scientific advances to promote early recognition, standardize diagnosis and treatment, and advance clinical and scientific practice.

Mesinovic J (2019) published “Sarcopenia and Type 2 Diabetes Mellitus: A Bidirectional Relationship” [11], discussing the reciprocal relationship between T2DM and sarcopenia, in which insulin resistance, inflammation, oxidative stress, and vascular complications drive mutual progression. Nomura T's 2018 study “Diabetic Polyneuropathy is a Risk Factor for Decline of Lower Extremity Strength in Patients with Type 2 Diabetes” [25] exhibited the highest centrality. This analysis of 1,442 participants demonstrated that diabetic polyneuropathy (DPN) is an independent risk factor for significant declines in lower extremity strength (11–16.6%) among middle-aged and elderly T2DM patients, while regular exercise serves as a protective factor for muscle function.

Cluster analysis using CiteSpace generated 10 clusters with a Q value of 0.6915 and an S value of 0.8539, indicating a stable, rational, and reliable structure. The largest cluster (#0) was “nutritional status”, followed by “managing sarcopenia” (#1), “scoping review” (#2), and “molecular mechanism” (#3). Other notable clusters included “sarcopenic obesity”, “non-alcoholic fatty liver disease”, “reduced cortical thickness”, “Kamogawa-DM cohort”, “cardiometabolic disease”, and “frailty diabetes”. These clusters indicate that current hot topics focus on clinical manifestations and comorbidities of DM and sarcopenic obesity, underlying pathological mechanisms, and nutritional or intervention strategies.

The term “citation burst” refers to papers that receive a rapid increase in citations within a specific period [26]. In this study, CiteSpace identified 10 references with citation bursts (Fig. **4D**), with the red portion of the timeline indicating periods of significant burst activity. Studenski *et al.'s* (2014) article “The FNIH Sarcopenia Project: Rationale, Study Description, Conference Recommendations, and Final Estimates” [27] had the highest burst intensity (11.17). This study, integrating data from nine large-scale cohorts, established evidence-based clinical thresholds for grip strength (male <26 kg, female <16 kg) and BMI-adjusted lean body mass in the extremities (male <0.789, female <0.512), providing critical criteria for sarcopenia diagnosis and intervention. Another high-intensity burst (10.66) was Kalyani *et al.'s* “Age-Related and Disease-Related Muscle Loss: The Effect of Diabetes, Obesity, and Other Diseases” [28], which demonstrated that aging and chronic conditions such as DM and obesity synergistically accelerate muscle loss through multiple mechanisms. By addressing sarcopenia diagnostic criteria and bidirectional mechanisms with chronic diseases, these studies lay the foundation for future research targeting precise interventions.

### 3.6 Keyword Analysis

The keywords of the literature reflect the main content and research hotspots in the field, and a keyword network diagram was generated using CiteSpace (Fig. **5A**). A total of 450 keywords were identified, among which 17 keywords appeared more than 100 times. Table **7** lists the top 5 keywords by frequency and centrality; the top keywords were “sarcopenia”, “insulin resistance”, “skeletal muscle”, “prevalence”, and “older adults”, reflecting the main research directions of sarcopenia and DM. The centrality of “adipose tissue” was the strongest (0.11), suggesting that “adipose tissue” may be a hot research topic in recent years.

Keyword clustering is the formation of interconnected network clusters of keywords with similar research topics in a research field, which can better illustrate the current research topics in the field. The keywords were clustered and analyzed using CiteSpace, as shown in Figs. (**5B**-**D**). Generally, Q>0.3 means that the structure of the network graph obtained is significant. The network has a Q value of 0.4498, S value of 0.7374 indicating reasonable and significant clustering of keywords in the field related to DM and sarcopenia. A total of 10 clusters were generated: muscle mass, diabetes mellitus, body composition, physical activity, muscle atrophy, resistance training, older adult, glycaemic control, leucine, and artificial pancreas. The clustering timeline graph is shown in Fig. (**6A**), extending to 2024 in the clusters with sarcopenia and DM influencing factors (older adult, muscle mass, body composition, and physical activity), sarcopenia-related treatments (resistance training, leucine), and DM monitoring and treatment (glycaemic control, leucine, artificial pancreas), suggesting that these have been the subject of ongoing research.

Keyword explosiveness represents cutting-edge disciplines and dynamic changes in a field. Fig. (**6B**) lists the top 25 most explosive keywords, with the blue line representing the time interval and the red line representing the period of keyword explosiveness. It can be observed that “postmenopausal women” have the highest intensity (4.92) in the outbreak graph. Based on the time of appearance, it can be observed that research on the relationship between DM and sarcopenia focuses on the analysis of early pathological mechanisms (“insulin resistance”, “insulin sensitivity”, human skeletal muscle”) to clinical interventions (“resistance exercise”, “body mass index”) and population management (“body mass index”). and population management (“postmenopausal women”, “older men”). This reflects the field's progression from basic research to clinical applications (Fig. **7**).

### 3.7 DM and Sarcopenia Intersection Targets and PPI Network Diagram

Figs. (**7A** and **B**) show the Venn diagram of the intersection targets between DM and sarcopenia, as well as the protein-protein interaction (PPI) network diagram. The Venn diagram indicates that 97 genes are shared between the two conditions, suggesting a high degree of overlap in their mechanisms. Network topology analysis identified the top five core hub genes, ranked by degree value as INS, ALB, AKT1, IL6, and TNF. The insulin-related gene INS has been identified as a core gene, which aligns consistently with the appearance of the keyword “insulin resistance” in the bibliometric analysis, validating the intrinsic connection between research hotspots and potential molecular mechanisms. INS is a key molecule linking the two diseases [8], while AKT1 is an important signaling protein involved in insulin signaling pathways [29] and muscle synthesis [30].

### 3.8 KEGG and GO Functional Enrichment Analysis

Figs. (**7C** and **D**) show the enrichment analysis results of disease intersection genes. GO functional enrichment analysis results showed that differentially expressed genes were mainly enriched in biological processes (BP) such as gene expression regulation and activation of the PI3K/MAPK signaling pathway, localized in cellular components (CC) such as the extracellular space and endoplasmic reticulum lumen, and involved in molecular functions (MF) such as hormone activity, cytokine activity, and insulin receptor binding. KEGG pathways analysis indicated that the adipokine signaling pathway, longevity regulation pathway, and type 2 diabetes and insulin resistance pathway were significantly enriched. These molecular pathway findings provide a foundation for understanding the biological basis underlying research trends observed in the bibliometric analysis.

## 4 DISCUSSION

### 4.1 General Information on the Main Findings

As the global population ages at an accelerated pace, the prevalence of DM and sarcopenia has shown a significant upward trend, emerging as a critical public health issue. This study employs bibliometric and bioinformatics methods to systematically analyze research hotspots, trends, and common molecular mechanisms in the fields of DM and sarcopenia. The results indicate that the annual number of publications and citation frequencies in this field have shown a sustained upward trend from 2015 to 2024, fully reflecting the urgent need for research in this interdisciplinary field under the backdrop of an aging society. From a national/regional perspective, China leads in the number of publications (294), while the United States stands out in terms of academic influence, with the highest total number of citations (8,848) and the strongest overall link strength (122), highlighting its leading academic position in this field. Notably, the combined publication volume of China and Japan accounts for 46.3% of the total, a phenomenon that may be closely related to the large population bases and rapid aging processes of these two countries. Further analysis reveals that research priorities across different countries/regions align closely with molecular mechanisms. For instance, Japanese scholars (such as the Fukui team) extensively report in clinical and epidemiological studies how inflammatory factors (IL-6, TNF) and mitochondrial dysfunction drive diabetic sarcopenia [22], fully consistent with our PPI network results identifying IL6 and TNF as core genes. Chinese researchers, however, have predominantly focused on experimental studies involving natural products and drug interventions. Utilizing bioinformatics methods, they have repeatedly validated pivotal genes such as AKT1 [31], which are significantly enriched in the PI3K-Akt-mTOR signaling pathway—a finding resonating with our KEGG analysis.

Publication preferences in journals also reflect the interdisciplinary integration of mechanisms, nutrition, and clinical applications. As the leading journal in publications, *Nutrients* prominently highlights the pivotal role of nutritional interventions in this field, with content frequently involving adipokine signaling pathways and longevity regulatory pathways—highly consistent with our pathway enrichment results. Conversely, the *Journal of Cachexia, Sarcopenia and Muscle* focus on muscle atrophy mechanisms, directly corresponding to our identified muscle-related core genes (AKT1, TNF). Cross-disciplinary network analysis reveals that molecular/immunological studies are predominantly cited by genetic mechanism research, reflecting a deepening from phenotype to mechanism. Meanwhile, clinical medical findings are cited by both basic science and medical journals, demonstrating bidirectional knowledge flow. Institutional collaboration network analysis shows that Hospital Universitario de Getafe has the highest centrality index (0.31), indicating that the institution plays a crucial hub role in the international collaboration network. High-output authors include Fukui Michiaki, Hamaguchi Masahide, and Hashimoto Yoshitaka, among others, highlighting their significant contributions to the field.

Bioinformatics analysis further elucidated the common molecular basis between sarcopenia and DM. At the molecular mechanism level, the PPI network identified five core genes: INS, ALB, AKT1, IL6, and TNF. GO analysis revealed these genes primarily participate in gene expression and signal pathway regulation (BP), are located in the extracellular region (CC), and possess hormone and growth factor activity (MF). KEGG identified 20 significant pathways, providing molecular evidence for comorbidity mechanisms. Research themes evolved over time: initial focus on “insulin resistance” (corresponding to INS as a core gene) gradually shifted toward intervention strategies like “resistance exercise” and “leucine”, corresponding to the role of pathways such as mTOR in muscle synthesis.

### 4.2 Early Research in the Field of DM and Sarcopenia to Analyze the Pathological Mechanisms and Clinical Phenotypes

The keywords “insulin resistance” and “human skeletal muscle” in the exploded diagram suggest that early research in this field focused on the molecular pathogenesis of DM and sarcopenia. “Insulin resistance” ranked second in keyword frequency with 212 occurrences. Chronic low-grade inflammation emerged as the primary driver of insulin resistance [32]. The PPI network diagram reveals that inflammatory cytokines IL-6 and TNF simultaneously function as core hub genes. Insulin resistance, as a central connecting mechanism between sarcopenia and DM, establishes a bidirectional relationship between the two through multiple pathophysiologic pathways. Studies have shown that insulin resistance, as one of the major drivers of DM [33], decreases skeletal muscle protein synthesis and increases catabolism in a state of insulin resistance [34], while the activity of mTOR signaling pathway is inhibited [35], mitochondrial density and oxidative capacity are significantly reduced, forming a vicious cycle; at the same time, chronic low-grade inflammation induced by persistent hyperglycemia results in the up- regulation of inflammatory factors, such as TNF-α and IL-6, which not only induce impaired insulin signaling, but also directly activate muscle catabolism [36, 37]; and the reduction of muscle, as the main glucose-processing organ, directly leads to decreased insulin sensitivity [38]. The keyword “adipose tissue” has the highest centrality, indicating that adipose tissue has become a research hotspot in this field. Its prominent position in bibliometrics is validated at the molecular level through the significant enrichment of the “adipocyte cytokine signaling pathway” in KEGG analysis. It has been found that excessive deposition of adipose tissue disrupts the body's metabolic homeostasis and leads to metabolic abnormalities in skeletal muscle [39], and Schrauwen-Hinderling *et al*. demonstrated by magnetic resonance spectroscopy that there is a significant positive correlation between intramuscular lipid deposition (IMCL) and insulin resistance [40], and that this “intramuscular fat infiltration” is a key pathologic mechanism linking sarcopenia and DM. These studies on bidirectional mechanisms of sarcopenia and DM provide a theoretical basis for combined interventions.

Then, with the harmonization of diagnostic criteria for sarcopenia in 2019 [24] and the emergence of standards of care for DM [41] research shifted to clinical phenotypic associations and co-morbidity analyses. Clustering of co-citations showed that “sarcopenic obesity” and “non-alcoholic fatty liver disease” became hotspots, suggesting a shift toward research on comorbidities in the area of DM and sarcopenia. As a specific clinical phenotype, sarcopenic obesity has unique metabolic characteristics and prognostic risks, with patients experiencing decreased muscle mass and function while maintaining or increasing adipose tissue mass, which further exacerbates insulin resistance and glucose metabolism disorders [42]. On the other hand, studies on the association of NAFLD, a common complication of DM, with sarcopenia suggest that hepatic steatosis may accelerate skeletal muscle proteolytic metabolism and inhibition of myo-satellite cell activity by promoting the release of inflammatory factors and increased oxidative stress [43, 44].

Recent longitudinal cohort studies have further revealed that the occurrence and disease progression of sarcopenia in diabetic patients present diverse clinical phenotypes, which can be stratified according to the degree of muscle mass loss, the pattern of muscle strength decline, and the characteristics of functional impairment [45]. Notably, there was significant heterogeneity in the response to interventions in patients with different clinical phenotypes, which provides an important basis for individualized treatment strategies. In addition, several data have shown that patients with DM combined with sarcopenia have a higher incidence of cardiovascular disease than patients with sarcopenia alone [46], as well as a higher risk of infections [47], underscoring the clinical severity of this composite pathology.

### 4.3 Population Management of Diseases

The occurrence of “old adult” in the keyword clustering and “old men” and “age” in the keyword outbreak map suggests that population aging is closely related to metabolic diseases. Research has proven that age is an important factor influencing DM and sarcopenia [48, 49]. The two often coexist and exacerbate each other in the elderly population; the elderly population can co-exacerbate DM and sarcopenia through hormonal changes, inflammation, lifestyle, and other pathways [5, 50]. Several cross-sectional studies have shown that the prevalence of sarcopenia in diabetic patients over 65 years of age is 30-45%, which is significantly higher than that of non-diabetic people of the same age [51].Therefore, the International Clinical Hypomuscular Disease Practice Guidelines recommend that diabetic patients over 65 years of age be included in routine screening for hypomuscular disease and that muscle mass and function be assessed at least once a year [52]. The highest intensity (4.92) was observed for “postmenopausal women”, which was associated with “bone mineral density” and “fracture risk”, reflecting the synergistic effect of estrogen deficiency on muscle atrophy and bone metabolism. This phenomenon suggests that special strategies should be formulated for postmenopausal women in clinical management, and that osteoporosis assessment and sarcopenia screening should be integrated into the chronic disease management system [53]. Future studies should further explore the differences in response to various types of interventions in different age and gender groups to provide stronger evidence-based support for disease population management.

### 4.4 Clinical Interventions and Nutritional Therapy

In recent years the focus of research has shifted to therapeutic strategies for the disease, and current knowledge suggests that nutritional interventions and exercise therapy are the primary treatment strategies for both DM and sarcopenia [24, 54], This is supported by the presence of “leucine” in the clustering diagram and “Vitamin D” and “resistance exercise” in the bursting diagram. These intervention keyword bursts receive mechanistic justification from our KEGG pathway enrichment, which shows “mTOR signaling pathway” and “AMPK signaling pathway” as significantly enriched. The precision and individualization of nutritional therapy is a hot topic in recent research. A randomized controlled trial of 150 elderly patients with DM combined with sarcopenia by Bauer *et al*. showed that daily supplementation with high-leucine protein combined with vitamin D significantly increased appendicular skeletal muscle index (ASMI) and improved SPPB scores compared with conventional protein supplementation [24, 55]. Exercise intervention, as another core therapeutic strategy, with optimal modalities and individualized protocols, has made important advances in recent years. The traditional view is that aerobic exercise primarily improves insulin sensitivity, whereas resistance training primarily increases muscle mass. However, a randomized controlled trial by Villareal *et al*. was the first to systematically compare the differential effects of different exercise modalities in elderly patients with DM combined with sarcopenia, and found that a mixture of training (resistance exercise combined with aerobic training) was advantageous compared to a single modality in terms of both improving glycated hemoglobin and increasing muscle mass [56]. DM combined with sarcopenia interventions are continuing to evolve, and future research should focus on precise intervention systems for patients to provide a more solid foundation for clinical practice.

The appearance of “artificial pancreas” in the clusters suggests that the emergence of precision medicine and new technologies has brought new research opportunities for DM combined with sarcopenia, with the rapid development of multi-omics technology and artificial intelligence, the future research direction of this field will be more focused on the analysis of the disease mechanism and the optimization of the precise intervention strategy.

### 4.5 Common Molecular Mechanisms of DM and Sarcopenia

This study validated the common roles of insulin signaling, chronic inflammation, and nutritional status in diabetes combined with sarcopenia by integrating PPI networks and bibliometric results. By comparing the INS as the central node in the PPI network with the frequently used keyword “insulin resistance” in bibliometrics, one can see the central role of insulin signaling in research. This highlights the crucial role of insulin dysfunction in the occurrence and development of diseases. Previous studies have shown that insulin resistance not only leads to abnormal glucose metabolism but also affects muscle protein synthesis by inhibiting the PI3K-Akt-mTOR pathway [57, 58]. The prominent positions of inflammatory factors IL-6 and TNF in the network further reveal the common role of chronic inflammation in the pathogenesis of both diseases. Chronic inflammation can simultaneously promote insulin resistance and muscle atrophy through multiple mechanisms [59, 60]. The importance of ALB is reflected in the alignment of molecular evidence with the keyword emphasis on “leucine”. The co-occurrence of ALB and inflammatory factors suggests the potential of the serum albumin/inflammatory marker ratio as a disease predictive indicator. In inflammatory responses, the pro-inflammatory cytokine TNF-α significantly reduces hepatic albumin synthesis capacity by inhibiting ALB mRNA transcription [61], leading to the occurrence of hypoalbuminemia. Hypoproteinemia not only indicates abnormal protein metabolism but is also closely associated with reduced muscle mass and impaired physical function [62]. In diabetic patients, serum inflammatory markers such as IL-6 and TNF-α are significantly elevated [63, 64], while ALB levels exhibit a negative correlation with inflammatory markers [65]. Additionally, as a biochemical indicator reflecting nutritional status, ALB levels are negatively correlated with all-cause mortality in community-dwelling elderly individuals at risk of sarcopenia [66].

KEGG pathway analysis revealed an enrichment of the adiponectin signaling pathway and confirmed that 'adipose tissue' has the highest keyword centrality (0.11). Adiponectin not only regulates insulin sensitivity [67] but also influences muscle metabolism through the AMPK pathway [68], providing a mechanistic basis for explaining the increased incidence of sarcopenia in diabetic patients. The enrichment of the mTOR and AMPK pathways validated the trends in intervention studies, particularly with the occurrence of “resistance exercise” and “leucine” in the keyword analysis [69, 70].

### 4.6 Clinical Significance and Translational Value of Research Findings

The identification of five core hub genes (INS, ALB, AKT1, IL6, TNF) provides important clues for understanding the molecular basis of diabetes with sarcopenia. The combined effects of insulin signaling pathway abnormalities, chronic inflammation, and malnutrition constitute the core mechanism for the decrease in muscle mass, and also provide a basis for precision therapy and biomarker development. The pivotal role of INS underscores the importance of improving insulin sensitivity as a core therapeutic direction. Recent studies indicate that the insulin sensitizer metformin significantly improves grip strength, walking speed, and short-term physical performance metrics in older adults [71]. The central roles of IL-6 and TNF confirm that chronic inflammation is not only a marker of disease progression but also a potential target for anti-inflammatory interventions. Inflammatory markers showed a significant reduction following combined exercise and nutritional interventions [72], supporting the clinical potential of lifestyle interventions. The prominence of ALB underscores the importance of nutritional support, particularly high-quality protein supplementation, as reflected in the emergence of keywords such as “resistance exercise” and “leucine” in bibliometric analyses. Overall, this study suggests that a combined exercise and nutritional intervention strategy has the greatest clinical translational value. IL-6, TNF-α, and ALB are not only potential biomarkers for early disease detection [65, 66, 73], but their ratios may predict disease progression and prognosis more accurately than single indicators. Future research should integrate multi-omics and metabolomics data to develop more precise diagnostic and predictive models, in order to promote individualized interventions.

### 4.7 Research Limitations

First, the data source is limited to the Web of Science Core Collection, which may miss important literature from other databases, such as Scopus or PubMed. Second, the language limitation (only English literature was included) may result in research results from non-English-speaking countries not being adequately reflected. Third, PPI and pathway enrichment analysis rely on existing databases, and the results still need further experimental verification. Future research needs to incorporate multi-database, multi-language literature and conduct relevant experiments to address these shortcomings.

## CONCLUSION

This research, which combines bibliometrics and bioinformatics, helps us gain a clearer understanding of the common mechanisms between diabetes and sarcopenia. The study found that hotspot directions (such as insulin resistance and adipose tissue) and molecular evidence (core genes INS, IL6, TNF) are highly consistent. We screened a total of 97 common acting genes, among which 5 core hub genes (INS, ALB, AKT1, IL6, TNF) are particularly critical and have potential therapeutic value. Enrichment analysis shows that the PI3K/MAPK signaling pathway and inflammation-related pathways are two important common mechanisms underlying the two diseases, providing direction for precision medicine. The development of the study has gradually expanded from molecular mechanisms to clinical manifestations and intervention methods, with a current focus on treatment plans based on molecular evidence. Through cross-validation of these two analytical methods, we are more confident in the clinical application of nutritional interventions, anti-inflammatory treatments, and insulin sensitization strategies. Future research should focus on the experimental validation of these results and promote the development of combined therapies targeting the identified molecular networks.

## Figures and Tables

**Fig. (1):**
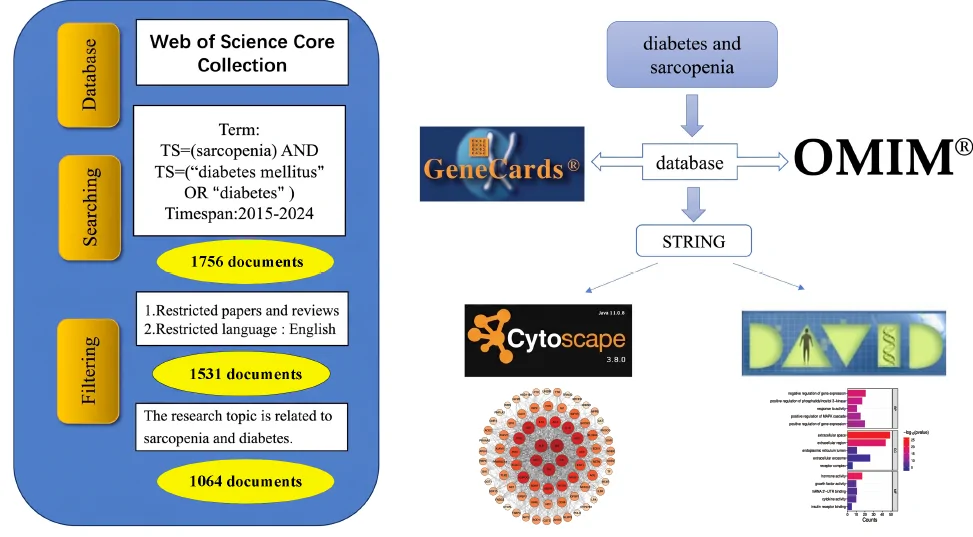
Search strategy and flowchart (Illustrates the systematic literature search process from Web of Science database, including inclusion/exclusion criteria application and the final selection of 1,064 publications for analysis).

**Fig. (2):**
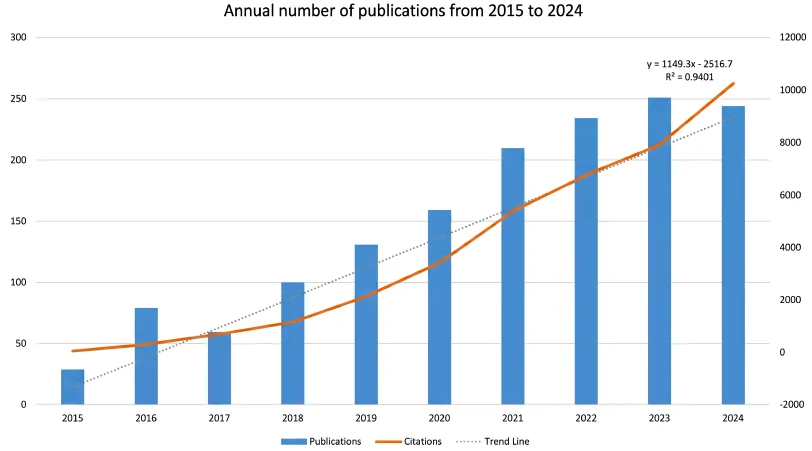
Annual number of publications from 2015 to 2014 (Demonstrates the temporal trend of diabetes and sarcopenia research publications, showing exponential growth from approximately 30 papers in 2015 to 250 papers in 2023).

**Fig. (3):**
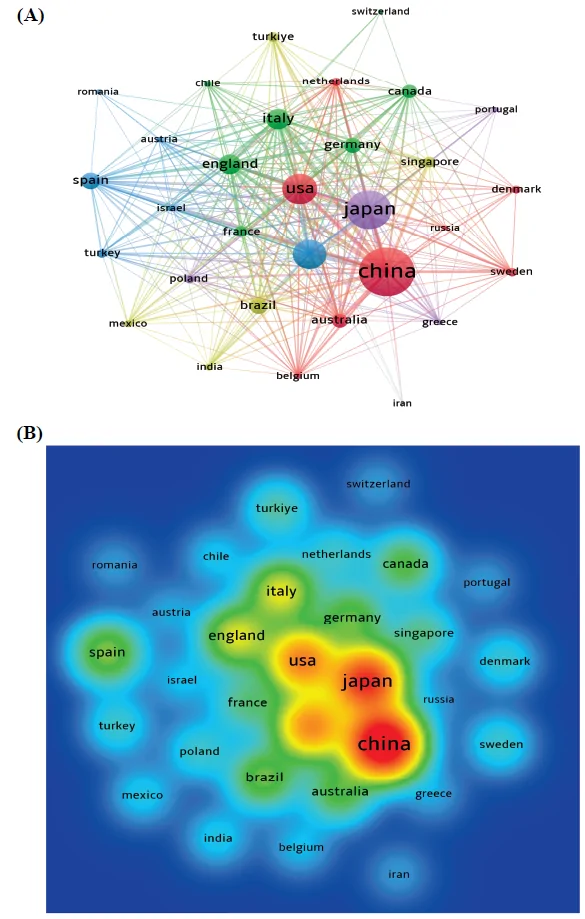
(**A**) Network visualization of international research collaborations (Visualization generated by VOSviewer. Each node represents a country. The size of the node reflects the frequency of keyword occurrences. The thickness of the connecting lines reflects the strength of the co-occurrence relationship between the two countries.); (**B**) Density map showing geographical distribution of research activity.

**Fig. (4):**
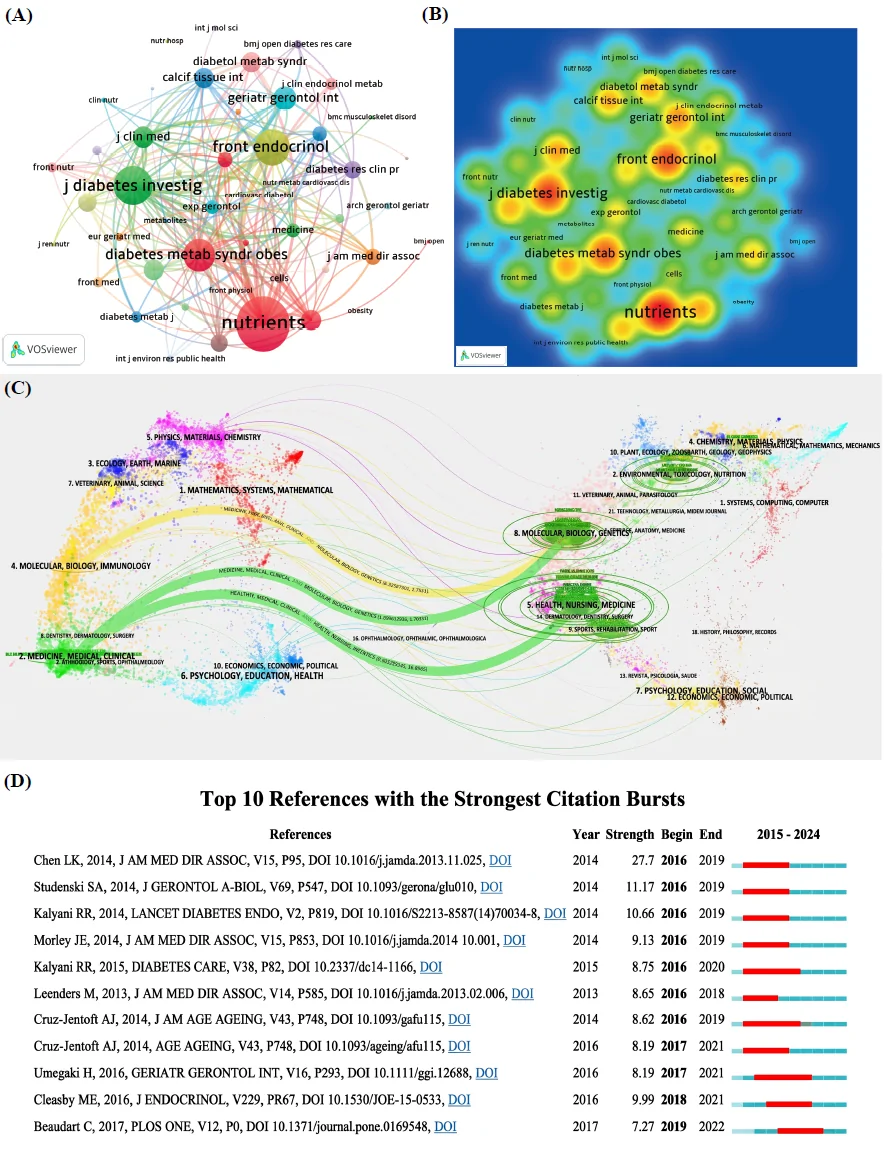
(**A**) Journal co-citation network showing interdisciplinary connections; (**B**) Journal density visualization indicating publication concentration; (**C**) Dual-map overlay revealing knowledge flow between disciplines; (**D**) Citation burst analysis of the top 10 most cited references.

**Fig. (5):**
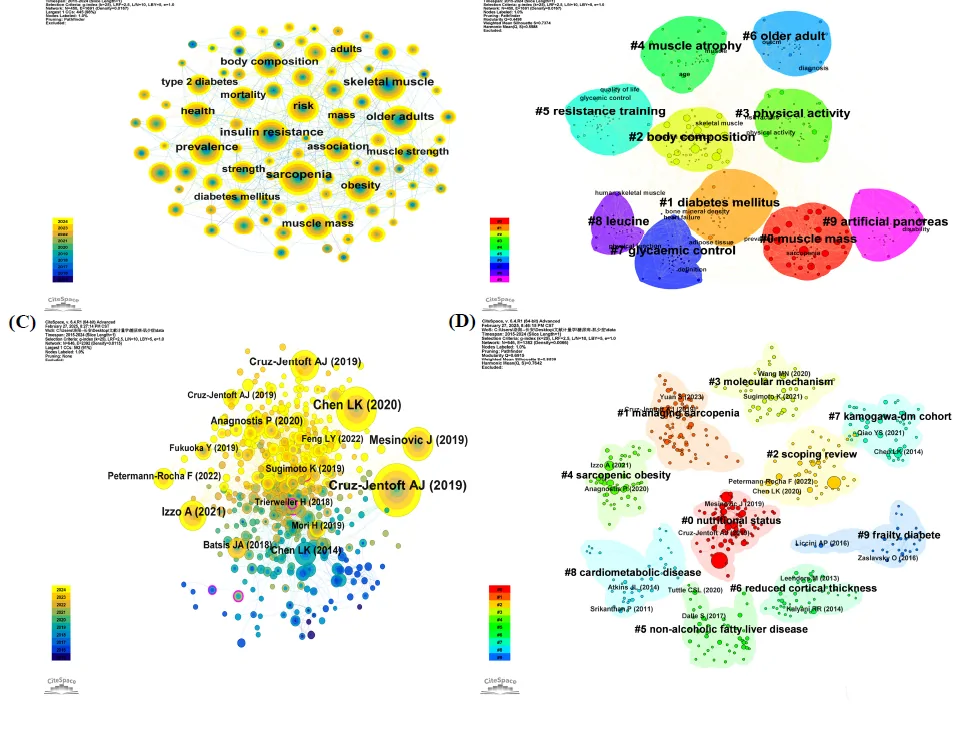
(**A**) Keyword co-occurrence network mapping research themes; (**B**) Keyword clustering showing thematic groupings; (**C**) Reference co-citation network; (**D**) Reference clustering revealing knowledge base structure (Colors indicate different clusters generated by CiteSpace/VOSviewer; node size corresponds to keyword frequency; connecting lines indicate co-occurrence relationships).

**Fig. (6):**
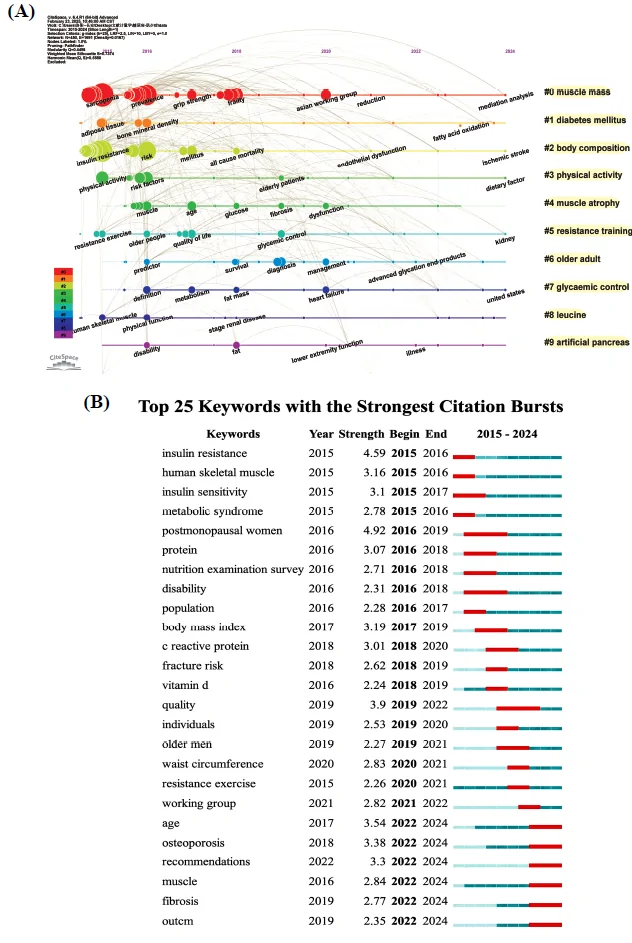
(**A**) Timeline view showing research theme evolution across three phases; (**B**) Top 25 keywords with strongest citation bursts, indicating emerging research hotspots.

**Fig. (7):**
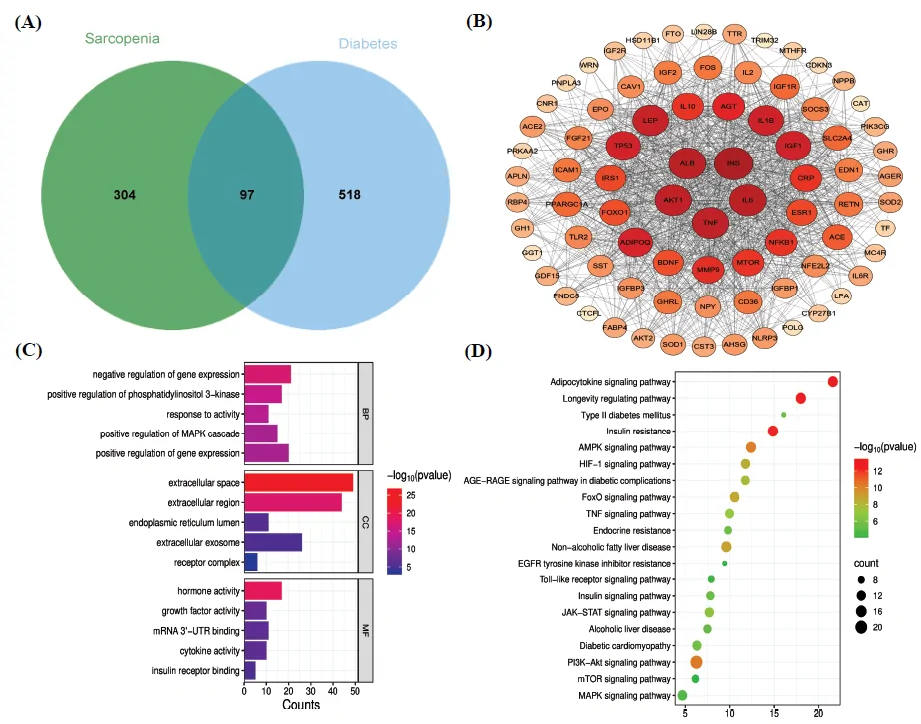
(**A**) Venn diagram displaying 97 shared target genes between diabetes and sarcopenia; (**B**) Protein-protein interaction network highlighting five core hub genes (INS, ALB, AKT1, IL6, TNF) with connection strengths (Node size represents degree centrality (number of connections); (**C**) Gene Ontology enrichment results for biological processes, cellular components, and molecular functions; (**D**) KEGG pathway enrichment analysis showing significantly enriched signaling pathways.

**Table 1:** Top 5 countries in terms of number of publications.

Rank	Country	Articles	Citations	Total Link Strength
1	China	294	5202	63
2	Japan	199	5133	37
3	USA	130	8488	122
4	South Korea	122	3343	39
5	Italy	66	5773	98

**Table 2:** The publication volume of the top 5 institutions.

Rank	Organization	Document
1	Kyoto Prefectural University of Medicine	27
2	Seoul National University	26
3	Yonsei University	25
4	Seoul National University Hospital	19
5	National University of Singapore	18

**Table 3:** Top 5 institutions in terms of centrality.

Rank	Organization	Centrality
1	Hospital Universitario de Getafe	0.31
2	National Center for Geriatrics & Gerontology	0.21
3	Kyoto University	0.19
4	Osaka University	0.17
5	Harvard University	0.17

**Table 4:** Top 5 authors in terms of publications.

Rank	Author	Document
1	Fukui, Michiaki	26
2	Hamaguchi, Masahide	25
3	Hashimoto, Yoshitaka	23
4	Okamura, Takuro	21
5	Yamazaki, Masahiro	20

**Table 5:** Top 5 journals in terms of publications.

Rank	Journal	Document
1	*Nutrients*	40
2	*Bmc geriatrics*	26
3	*Journal of Cachexia Sarcopenia and Muscle*	26
4	*Journal of Clinical Medicine*	23
5	*Journal of Diabetes Investigation*	23

**Table 6:** Top 5 references for centrality and frequence.

Rank	Co-cited Reference	Frequence	Rank	Co-cited Reference	Centrality
1	Cruz-Jentoft AJ (2019)	265	1	Nomura T (2018)	0.15
2	Chen LK (2020)	181	2	Trierweiler H (2018)	0.12
3	Mesinovic J (2019)	106	3	Chen FQ (2020)	0.11
4	Cruz-Jentoft AJ (2019)	89	4	Chiu CY (2016)	0.11
5	Izzo A (2021)	83	5	Cohen S (2015)	0.1

**Table 7:** Top 5 for keyword frequency and centrality.

Rank	Keyword	Frequency	Rank	Keyword	Centrality
1	sarcopenia	242	1	adipose tissue	0.11
2	insulin resistance	212	2	muscle mass	0.07
3	skeletal muscle	210	3	insulin	0.06
4	prevalence	187	4	human skeletal muscle	0.06
5	older adults	184	5	metabolic syndrome	0.05

## LIST OF ABBREVIATIONS

DM: Diabetes Mellitus
T1DM: Type 1 Diabetes Mellitus
T2DM: Type 2 Diabetes Mellitus
WOSCC: Web of Science Core Collection
PPI: Protein-Protein Interaction
KEGG: Kyoto Encyclopedia of Genes and Genomes
GO: Gene Ontology
BP: Biological Process
CC: Cellular Component
MF: Molecular Function
BC: Betweenness Centrality
DPN: Diabetic Polyneuropathy
NAFLD: Non-Alcoholic Fatty Liver Disease
IMCL: Intramuscular Lipid Deposition
ASMI: Appendicular Skeletal Muscle Index
SPPB: Short Physical Performance Battery
FNIH: Foundation for the National Institutes of Health
BMI: Body Mass Index
TNF: Tumor Necrosis Factor
IL-6: Interleukin-6
ALB: Albumin
INS: Insulin
AKT1: AKT Serine/Threonine Kinase 1
PI3K: Phosphoinositide 3-Kinase
MAPK: Mitogen-Activated Protein Kinase
mTOR: Mechanistic Target of Rapamycin
AMPK: AMP-Activated Protein Kinase
